# Regulation of the BMP Signaling-Responsive Transcriptional Network in the *Drosophila* Embryo

**DOI:** 10.1371/journal.pgen.1006164

**Published:** 2016-07-05

**Authors:** Lisa Deignan, Marco T. Pinheiro, Catherine Sutcliffe, Abbie Saunders, Scott G. Wilcockson, Leo A. H. Zeef, Ian J. Donaldson, Hilary L. Ashe

**Affiliations:** Faculty of Life Sciences, University of Manchester, Manchester, United Kingdom; Harvard Medical School, Howard Hughes Medical Institute, UNITED STATES

## Abstract

The BMP signaling pathway has a conserved role in dorsal-ventral axis patterning during embryonic development. In *Drosophila*, graded BMP signaling is transduced by the Mad transcription factor and opposed by the Brinker repressor. In this study, using the *Drosophila* embryo as a model, we combine RNA-seq with Mad and Brinker ChIP-seq to decipher the BMP-responsive transcriptional network underpinning differentiation of the dorsal ectoderm during dorsal-ventral axis patterning. We identify multiple new BMP target genes, including positive and negative regulators of EGF signaling. Manipulation of EGF signaling levels by loss- and gain-of-function studies reveals that EGF signaling negatively regulates embryonic BMP-responsive transcription. Therefore, the BMP gene network has a self-regulating property in that it establishes a balance between its activity and that of the antagonistic EGF signaling pathway to facilitate correct patterning. In terms of BMP-dependent transcription, we identify key roles for the Zelda and Zerknüllt transcription factors in establishing the resulting expression domain, and find widespread binding of insulator proteins to the Mad and Brinker-bound genomic regions. Analysis of embryos lacking the BEAF-32 insulator protein shows reduced transcription of a peak BMP target gene and a reduction in the number of amnioserosa cells, the fate specified by peak BMP signaling. We incorporate our findings into a model for Mad-dependent activation, and discuss its relevance to BMP signal interpretation in vertebrates.

## Author Summary

Embryogenesis involves the patterning of many different cell fates by a limited number of types of signals. One way that these signals promote a particular cell fate is through the induction of a complex, yet highly reproducible, gene expression programme that instructs changes in the cell. For example, there is a conserved role for BMP signals in specifying cell fates during dorsal-ventral axis patterning. Here, we have used genomics approaches to identify the gene expression programme implemented in response to BMP signaling during axis patterning in the *Drosophila* embryo. Part of the gene network downstream of BMP signaling includes members of the EGF signaling pathway, with our data highlighting reciprocal interactions between these two pathways. We have also determined genome-wide binding of BMP-responsive transcription factors to gain new insights into how the BMP gene network is activated. Our data reveal roles for specific transcription factors and insulator binding proteins, with the latter traditionally associated with the separation of transcriptional domains. Overall, our data will provide a platform for exploiting the tractability of the *Drosophila* embryo to determine which features of the network are critical drivers of BMP-induced cell fate changes during embryogenesis.

## Introduction

The Bone Morphogenetic Protein (BMP) signaling pathway is used repeatedly throughout development to regulate a diverse array of processes. One of the major conserved roles of the BMP pathway is to specify epidermal fates during embryonic dorsal-ventral axis patterning [1]. For example, in *Drosophila*, a heterodimer of the BMP signaling molecules, Decapentaplegic (Dpp) and Screw (Scw), forms a gradient in order to pattern the dorsal ectoderm of the embryo into dorsal epidermis and amnioserosa fates. Dpp-Scw signaling through a Thickveins (Tkv), Punt and Saxophone receptor complex results in phosphorylation of the C-terminal tail of the Mothers Against Dpp (Mad) transcription factor (pMad), which then interacts with Medea (Med), leading to their stabilization in the nucleus [2].

Initially, nuclear pMad is observed around the dorsal midline following extracellular transport of the Dpp-Scw ligand as the first step in gradient formation [2]. The pMad distribution is then refined to a narrower stripe by positive feedback via transcription of Dpp-Scw target genes, including that of *eiger* (*egr*), which encodes a TNF-α ligand [3]. Studies in flies and vertebrates have revealed complex regulation of Smad transcription factors by phosphorylation, dephosphorylation, SUMOylation and ubiquitination, which controls their subcellular localization, transcriptional activity and degradation [1, 4].

Mad and Med both activate and repress transcription [5]. Smad-dependent activation can be attenuated by the Brinker (Brk) repressor, which is expressed in the neuroectoderm underlying the dorsal ectoderm [6]. Evidence has been obtained for competitive binding between Mad and Brk at Dpp-responsive enhancers during different developmental stages [5]. In terms of Mad-Med activation, DNA binding motifs have been identified from which loose consensus sequences have been derived [7]. In addition, a well-characterized, conserved Activation Element has been described, along with a related Silencer Element that mediates Dpp-dependent repression via recruitment of the Schnurri (Shn) corepressor [5].

Genomics approaches have facilitated identification of developmental gene networks in the *Drosophila* embryo, particularly those activated by the Dorsal transcription factor or underpinning mesoderm patterning in the embryo [8, 9]. Despite progress in deciphering the Dorsal gene regulatory network during early patterning of the embryo, details relating to the transcription network underpinning dorsal ectoderm differentiation in response to Dpp are sparse. In this study, we investigate the transcriptional responses downstream of the major developmental BMP signaling pathway in the *Drosophila* embryo. Our data identify multiple target genes and enhancers, and reveal that the EGF pathway activity is constrained in the embryo to facilitate correct Dpp-dependent patterning. In addition, we show roles for Zelda (Zld), Zerknüllt (Zen) and the BEAF-32 insulator protein in Dpp gradient interpretation.

## Results

### Identification of genome-wide targets of embryonic Dpp signaling

**Fig 1:**
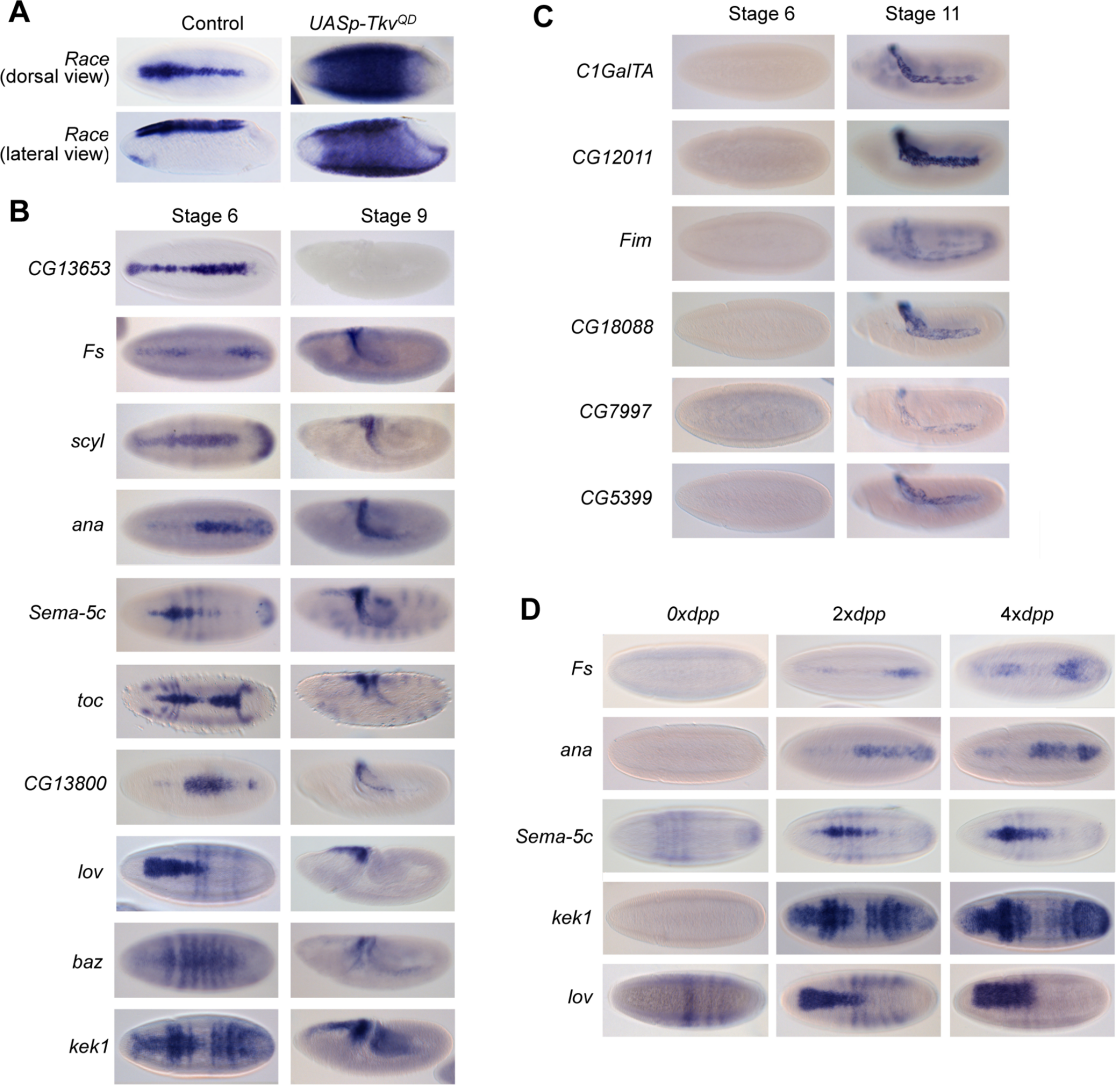
Identification of Dpp target genes. (A) *Race* expression in dorsal and lateral views of wildtype and *Tkv*^*QD*^ over-expressing embryos at the onset of gastrulation. (B) RNA *in situ* hybridization staining of wildtype embryos with antisense probes to detect transcripts identified as being positively regulated by Dpp signaling, based on the RNA-seq analysis. Dorsal and lateral views of stage 6 and 9 embryos, respectively, are shown. (C) As in (B), except stage 6 and 11 embryos are shown. (D) Expression of selected transcripts in embryos lacking *dpp* or carrying 4 copies of *dpp*, compared to the wildtype pattern.

We used RNA-seq to identify the Dpp signaling responsive transcription network in the early *Drosophila* embryo. We ectopically activated Dpp signaling in the embryo by ubiquitously expressing the constitutively activated *Tkv* receptor (*UASp-Tkv*^*QD*^) [10] using a maternal GAL4 driver, which leads to expanded expression patterns of Dpp target genes, including *Race* (Fig 1A). mRNA levels in these *Tkv*^*QD*^ expressing embryos were compared to control embryos carrying only the GAL4 driver, at 3–3.5 h after egg laying (AEL) when there is robust Dpp target gene transcription. We identified 331 genes (excluding *w* and *tkv* which are present in the *UASp-Tkv*^*QD*^ transgene) with altered expression patterns in the *Tkv*^*QD*^ versus control embryos at the chosen statistical cut-off, of which 109 are upregulated and 222 downregulated (S1 Dataset). These include 12 out of 14 previously identified Dpp target genes (S1 Dataset), with the Dpp targets *rhomboid* (*rho*) and *pannier* (*pnr*) below the cut-off. Validation of a subset of positively regulated Dpp target genes revealed a range of different expression patterns in the dorsal ectoderm (Fig 1B). Some of the targets show amnioserosa specific expression in later stage embryos, without earlier expression (Fig 1C). Confirmation that a subset of genes identified are authentic Dpp targets comes from their loss of expression in *dpp* mutant embryos, with a concomitant expansion in embryos with 4 copies of *dpp* (Fig 1D). We also mined the Flyexpress collection of *Drosophila* embryo RNA *in situ* hybridization data [11] for the remaining upregulated Dpp target genes, which together with our expression pattern analysis revealed that ~40% of the genes with a known expression pattern (ie 33 of 84) show restricted expression in the dorsal ectoderm of the early embryo as expected for early Dpp target genes (S1 Dataset). This proportion rises to ~70% when gene expression patterns consistent with being activated by Dpp later in embryogenesis are included (eg Fig 1C). Some of the false positives may be due to transcripts upregulated during oogenesis by the maternally driven *Tkv*^*QD*^ receptor.

One known Dpp target gene missed by our RNA-seq data is *pnr*, a gene expressed throughout the dorsal ectoderm in the central region of the embryo. Less expansion of broad targets is predicted in *Tkv*^*QD*^ overexpressing embryos compared to peak Dpp targets, and we note that our RNA-seq approach is more biased towards identification of peak or intermediate Dpp target genes (Fig 1B). In addition, we find that *pnr* is repressed in the mesoderm in the *Tkv*^*QD*^ overexpressing embryos, consistent with the Snail repressor binding to its enhancer [12]. Therefore, we also searched the Flyexpress data [11] for expression patterns similar to known Dpp target genes. This analysis identified a further 32 potential Dpp targets (S1 Dataset). Taking the Dpp target genes from these two approaches together, analysis of GO terms revealed categories consistent with biological processes known to be activated, eg dorsal closure and amnioserosa maintenance, and repressed, eg nervous system development, by Dpp during embryogenesis (S1 Fig) [13, 14].

### Enhancer identification by pMad and Brk ChIP-seq

**Fig 2:**
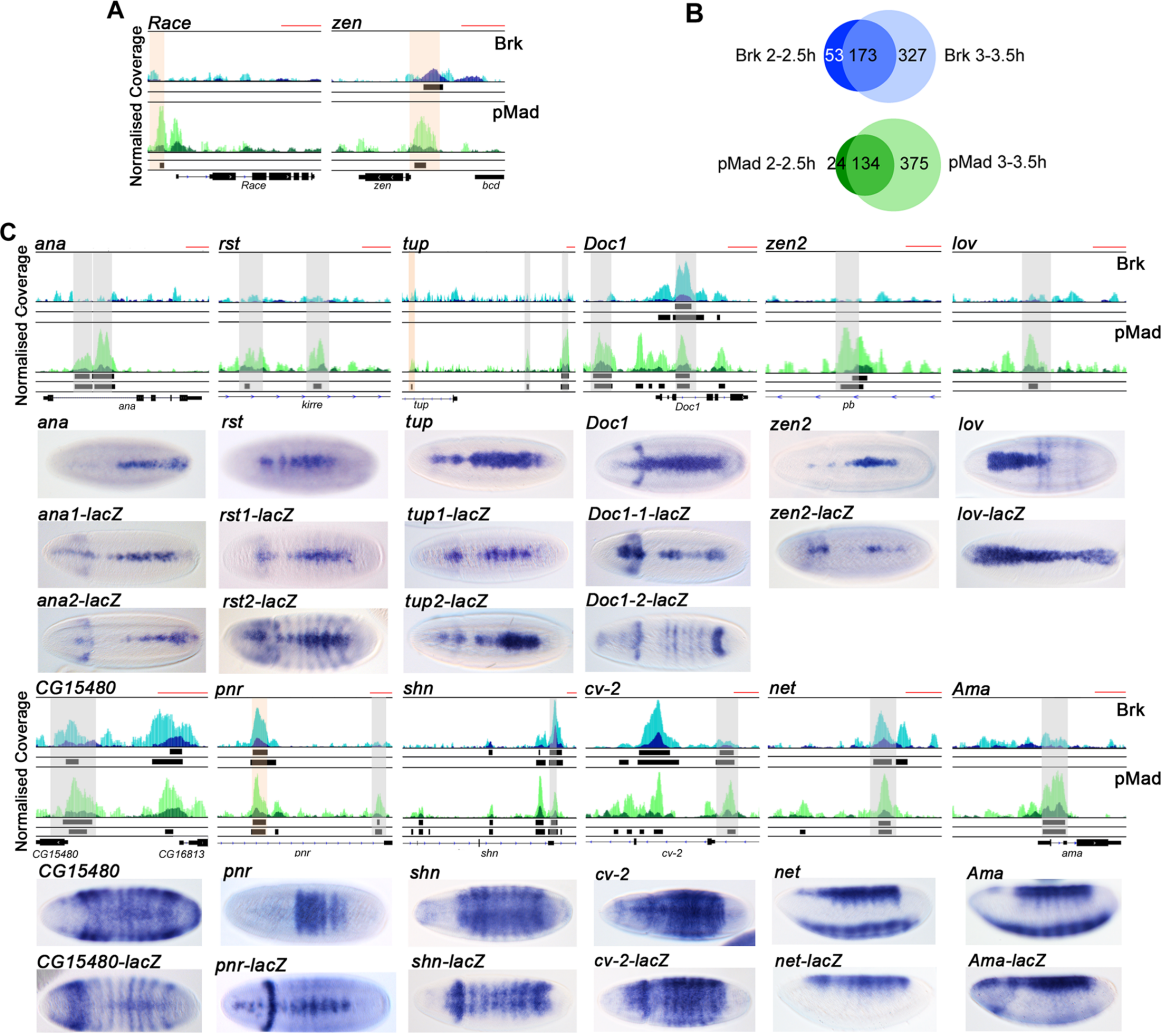
pMad and Brk ChIP-seq validation. (A) Browser images of ChIP-seq reads normalized to the PI control for pMad (dark green, 2–2.5 h, light green, 3–3.5 h AEL) and Brk (dark blue, 2–2.5 h, light blue, 3–3.5 h AEL) for *Race* and *zen*. Called peaks are shown as black bars below the Brk and pMad tracks (top row 2–2.5 h, bottom row 3–3.5 h). The red scale bar at the top right of each browser view represents 1kb. Known enhancers are shaded in orange. (B) Venn diagrams showing the overlap between the data sets for each factor between the two time points. (C) Browser views as in (A) for putative enhancers based on pMad/Brk binding. In situ hybridizations below the browser views show the endogenous expression pattern for the indicated gene, and the *lacZ* reporter gene expression pattern driven by the test ChIP region. Embryos are dorsal views with the exception of the *net* and *Ama* embryos, which are shown as lateral views to highlight that the test region only drives the dorsal ectoderm part of the expression pattern. Grey shaded areas indicate the tested enhancer fragments, orange shading indicates a known enhancer. Where two are tested, the regions are labelled 1 and 2 as seen from left to right. The enhancer for *zen2* is located within the *pb* gene. The absence of a gene structure below the tracks reflects distal enhancer positioning.

To investigate regulation of the Dpp-responsive network, we identified Dpp-responsive enhancers by performing pMad and Brk ChIP-seq on wildtype embryos at 2–2.5 h and 3–3.5 h AEL, corresponding to the initial activation and maintenance of Dpp-dependent transcription, respectively. Two biological replicates were sequenced for each factor and associated control at each time point. We used a high stringency cut-off to define pMad and Brk binding regions (see Methods), and these included enhancers for known target genes, such as *Race*, *zen*, *tailup (tup)* and *pannier* (*pnr)* (Fig 2A and 2C, S2 Dataset) [12, 15, 16].

At 2–2.5 h, with the selected cut-off, we identified 226 binding regions for Brk and 158 binding regions for pMad (Fig 2B). At 3–3.5 h we detect at least double the number of binding regions for both factors, with each binding around 500 sites (S2 Dataset). For both pMad and Brk, at least three quarters of the binding regions present at 2–2.5 h are also present at 3–3.5 h, suggesting that Brk and pMad occupy similar locations at 3–3.5 h as 2–2.5 h, with additional binding to other enhancers at the later time point (Fig 2B). Of the regions bound by pMad, 26 and 96 are also bound by Brk at 2–2.5h and 3–3.5h, respectively (S2 Dataset).

We next used transgenic reporter assays to validate some of the putative pMad/Brk responsive enhancers. The following regions recapitulated the endogenous expression pattern of an adjacent gene in the majority of cases: two *tup* enhancers, in addition to the *tup* enhancer identified previously [12], two enhancers each for *anachronism (ana)* and *roughest (rst)*, and a single enhancer for *zerknullt-related (zen2)*, *CG15480*, *shn*, *crossveinless 2* (*cv-2)*, *net* and *Amalgam (Ama)* (Fig 2C). In contrast to the endogenous *jim lovell (lov)* expression pattern, the *lov* enhancer also drives expression in the posterior of the embryo suggesting that a binding site for a repressor is absent, whereas the *cv-2* enhancer directs expression in the dorsal ectoderm, but lacks the peak of expression at the midline. One of the putative *Dorsocross1 (Doc1)* enhancers tested drives expression similar to that of *Doc1*, whereas the other directs broader, dorsal expression. Finally, the *pnr* enhancer tested results in a narrower expression than the *pnr* gene (Fig 2C). Overall, this validation confirms that the pMad/Brk bound regions drive restricted expression patterns in the dorsal ectoderm.

Integration of the RNA-seq and ChIP-seq data sets revealed that ~30% of ChIP peaks have a differentially expressed gene within 40kb (S2Ai Fig, S3–S5 Datasets), with genes potentially regulated by multiple enhancers, as validated for a subset above (Fig 2C). Overall genes in the differentially expressed set are significantly more likely to be located near to a ChIP peak than a gene that is not differentially expressed (S2Aii Fig). In addition, ~70% of the 65 Dpp target genes confirmed to be upregulated in the early embryo (S1 Dataset) have a ChIP peak within 40kb (S2B Fig, S3–S5 Datasets). Together these data suggest that the majority of positive Dpp targets in the embryo are directly activated by pMad. As Dorsal, Twist and Snail (DTS) binding to some Dpp target gene enhancers has been reported previously [12], we integrated the DTS dataset with our ChIP-seq data to address this question on a larger scale. This analysis revealed that ~20% of the Brk and pMad ChIP peaks are also bound by DTS (S2C Fig, S2 Dataset), consistent with dual regulation by DTS and Dpp signaling along the DV axis.

### Dpp-EGF crosstalk in the early embryo

Analysis of the RNA-seq data revealed enrichment of EGF signaling pathway members, including *rho* and *kekkon-1 (kek1)*, which are positive Dpp targets and *epidermal growth factor receptor* (*egfr)* that is a negative Dpp target (Fig 1B and 1D, S1 Dataset). In addition, the ChIP-seq data identify *egfr* and *argos* (*aos)*, encoding an EGF ligand inhibitor, as both pMad and Brk targets, with *vein*, encoding an EGF ligand, also a pMad target (S2 Dataset). In situ hybridization revealed that *egfr* and *aos* are restricted to the dorsal ectoderm by Brk repression (S3A Fig), and also repressed by peak Dpp signaling at the dorsal midline, whereas both *rho* [17] and *vein* are positive targets so their expression is lost in *dpp* mutants (S3B Fig).

**Fig 3:**
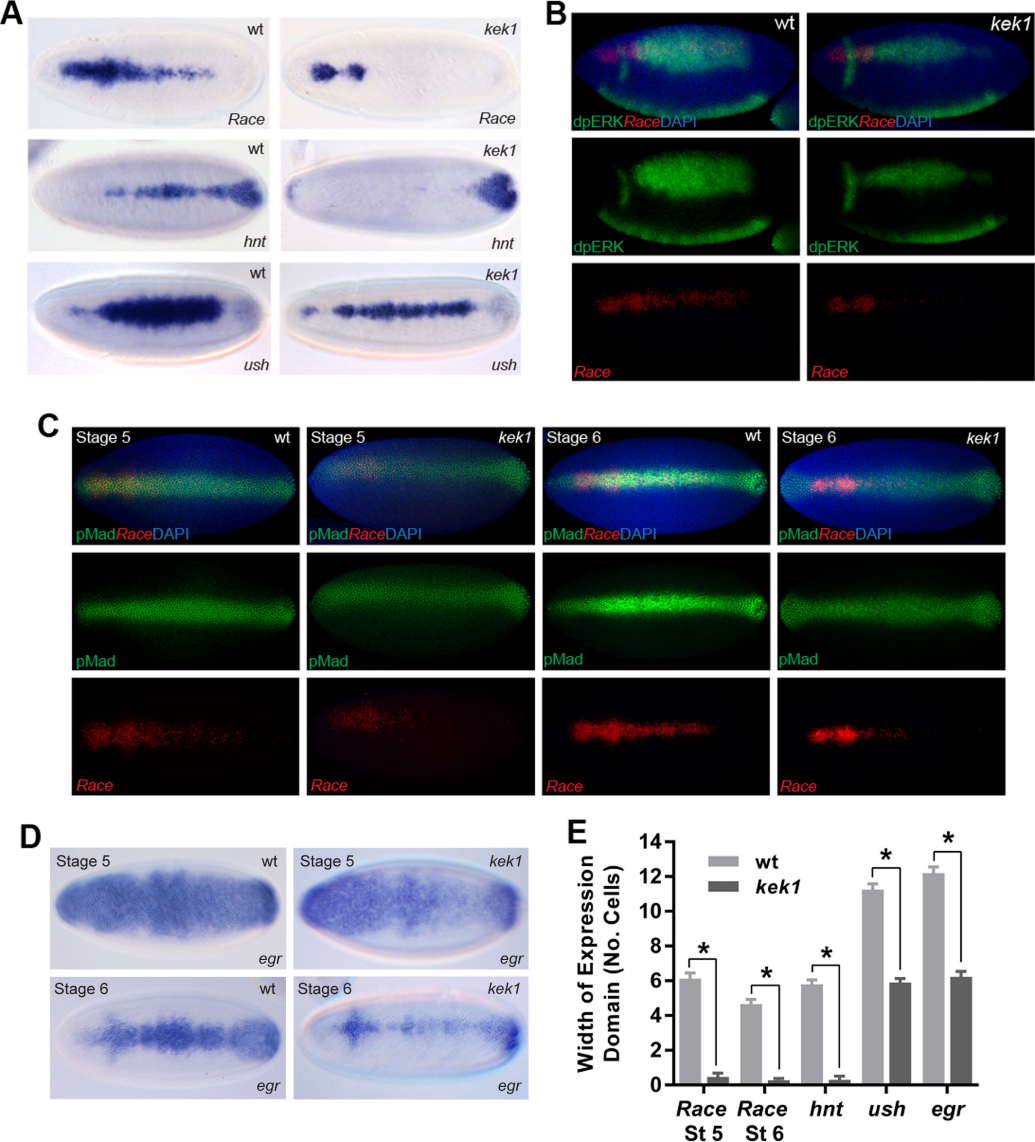
Dpp-EGF signaling cross-talk in the embryo. (A) RNA in situ hybridization showing expression of the peak Dpp target genes *Race*, *hnt* and *ush* in stage 6 wildtype and *kek1* mutant embryos (dorsal views). (B) Visualization of *Race* mRNA by fluorescent in situ hybridization (red) and dpERK protein (green) in wildtype and *kek1* mutant embryos. Top row shows the merge image with DAPI (blue), lower panels show the separate channels for *Race* and dpERK. (C) As in (B) except that *kek1* and wildtype embryos are stained for *Race* mRNA and pMad protein at stages 5 and 6, as labelled. (D) RNA in situ hybridizations showing *egr* mRNA in stage 5 and 6 wildtype and *kek1* mutant embryos. (E) Graph showing the expression widths of the Dpp target genes in wildtype and *kek1* mutant embryos. Error bars are SEM, n≥10 across different biological repeats, *P<0.0001, two-way ANOVA.

To investigate potential cross-talk between Dpp and EGF signaling, we analyzed Dpp target genes in embryos mutant for *kek1*, which encodes an inhibitor of EGF ligand-receptor interactions [18]. These embryos show disrupted expression of the peak Dpp target genes *Race*, *hindsight (hnt)* and *u-shaped (ush)* (Fig 3A and 3E). We next visualized diphosphorylated ERK (dpERK), which is activated by EGF signaling, in *kek1* and wildtype embryos. In wildtype embryos, dpERK is activated in a broad domain across *rho/vein* expressing cells (Fig 3B). In contrast, in *kek1* mutant embryos, dpERK is present in a narrower domain (Fig 3B), consistent with increased receptor activity sequestering the ligand. We also visualized pMad expression, which revealed a similar domain of activated pMad in stage 5 wildtype and *kek1* mutant embryos, although the latter embryos show weaker staining intensity (Fig 3C). Moreover, at stage 6 while the pMad staining refines into a narrower, more intense stripe in wildtype embryos as described previously [3], this refinement is not observed in the *kek1* mutant embryos (Fig 3C). As refinement of the pMad stripe is a result of positive feedback via Dpp target genes such as *egr* [3], we also visualized *egr* staining in the *kek1* mutant embryos. As shown in Fig 3D and quantitated in Fig 3E, *egr* staining is weaker in both stage 5 and 6 *kek1* embryos compared to wildtype. Together these data suggest that in the absence of Kek1, the lower pMad in stage 5 embryos leads to loss of positive feedback, resulting in a further reduction in the pMad peak at stage 6, and disrupted Dpp target gene expression.

**Fig 4:**
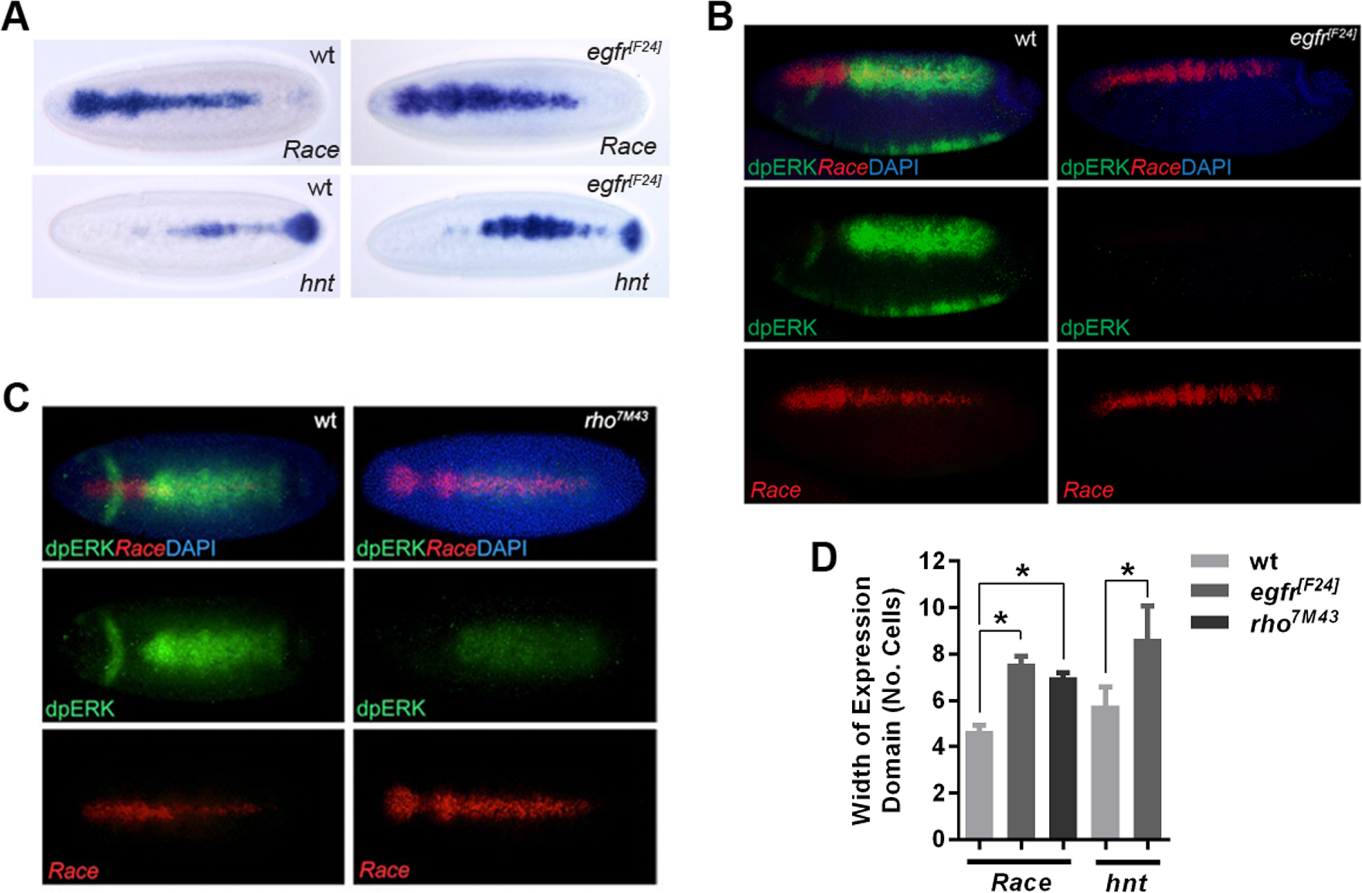
Negative regulation of the Dpp pathway by EGF signaling. (A) Visualization of *Race* and *hnt* mRNA in stage 6 wildtype and *egfr* mutant embryos by RNA in situ hybridization. (B) Detection of *Race* mRNA by fluorescent RNA in situ hybridization (red) and dpERK protein (green) by immunostaining in wildtype and *egfr* mutant embryos (late stage 6, dorsolateral views). (C) As in (B) except that *rho* mutant embryos are compared to wildtype, embryos are at early stage 6 and shown as dorsal views. (D) Graph showing the expression widths of the Dpp target genes in wildtype embryos and the mutants tested in (A)-(C). Error bars are SEM, n≥10 across different biological repeats, *P<0.0001, ordinary one-way ANOVA.

We also analyzed embryos with reduced EGF signaling. In *egfr* mutant embryos, the *Race* and *hnt* expression patterns are broader, particularly in the central region of the embryo (Fig 4A and 4D). Visualization of dpERK and *Race* expression confirms the loss of dpERK in the absence of EGFR activity (Fig 4B). In addition, *rho* mutant embryos show a weak patch of dpERK, consistent with pathway activation only by the Vein ligand, and expanded *Race* expression (Fig 4C and 4D). Together these gain- and loss-of-function data support negative regulation of Dpp pathway activation by EGF signaling.

### Motif analysis and the role of Zen

**Fig 5:**
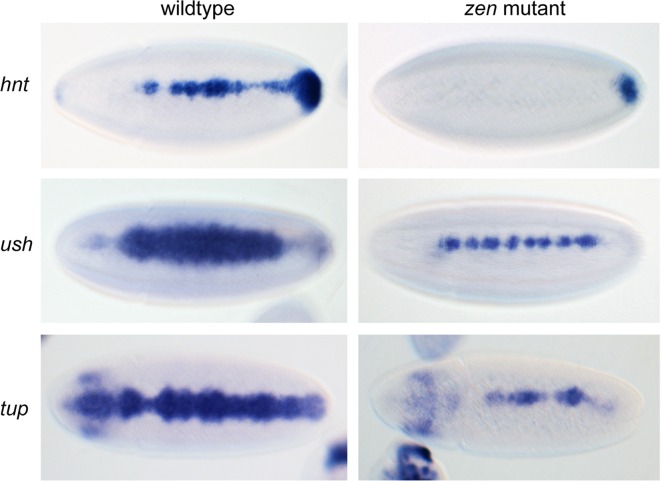
Peak and intermediate Dpp targets are disrupted in *zen* mutants. RNA in situ hybridization of stage 6 embryos (dorsal views) showing the expression patterns of the Dpp target genes *hnt*, *ush* and *tup* in *zen* mutant embryos compared to wildtype.

We next investigated the presence of Brk and Smad motifs in the pMad/Brk binding regions. The Mad [7], Med [5] and Brk [19] motifs are significantly enriched in the pMad-bound regions, compared to a set of housekeeping enhancers [20] (S4 Fig). We also performed *de novo* motif analysis using the set of enhancers we have validated (Fig 2C) and those characterized previously (*Race*, *zen*, *C15*, *tup*, *pnr*). We identified motifs carrying a TAAT core, consistent with the sites binding a homeodomain protein. Zen is a homeodomain protein that is required for activation of some Dpp target genes [3, 21, 22]. We extended these studies by visualizing the expression patterns of different Dpp threshold responses in *zen* mutant embryos. Expression of the peak Dpp target gene *hnt* is lost at the midline in *zen* mutant embryos (Fig 5). The intermediate Dpp target genes, *ush* and *tup*, also show thinner expression in *zen* mutants (Fig 5), suggesting that peak and intermediate Dpp targets require an input from Zen.

### Zld potentiates Dpp-dependent gene expression

**Fig 6:**
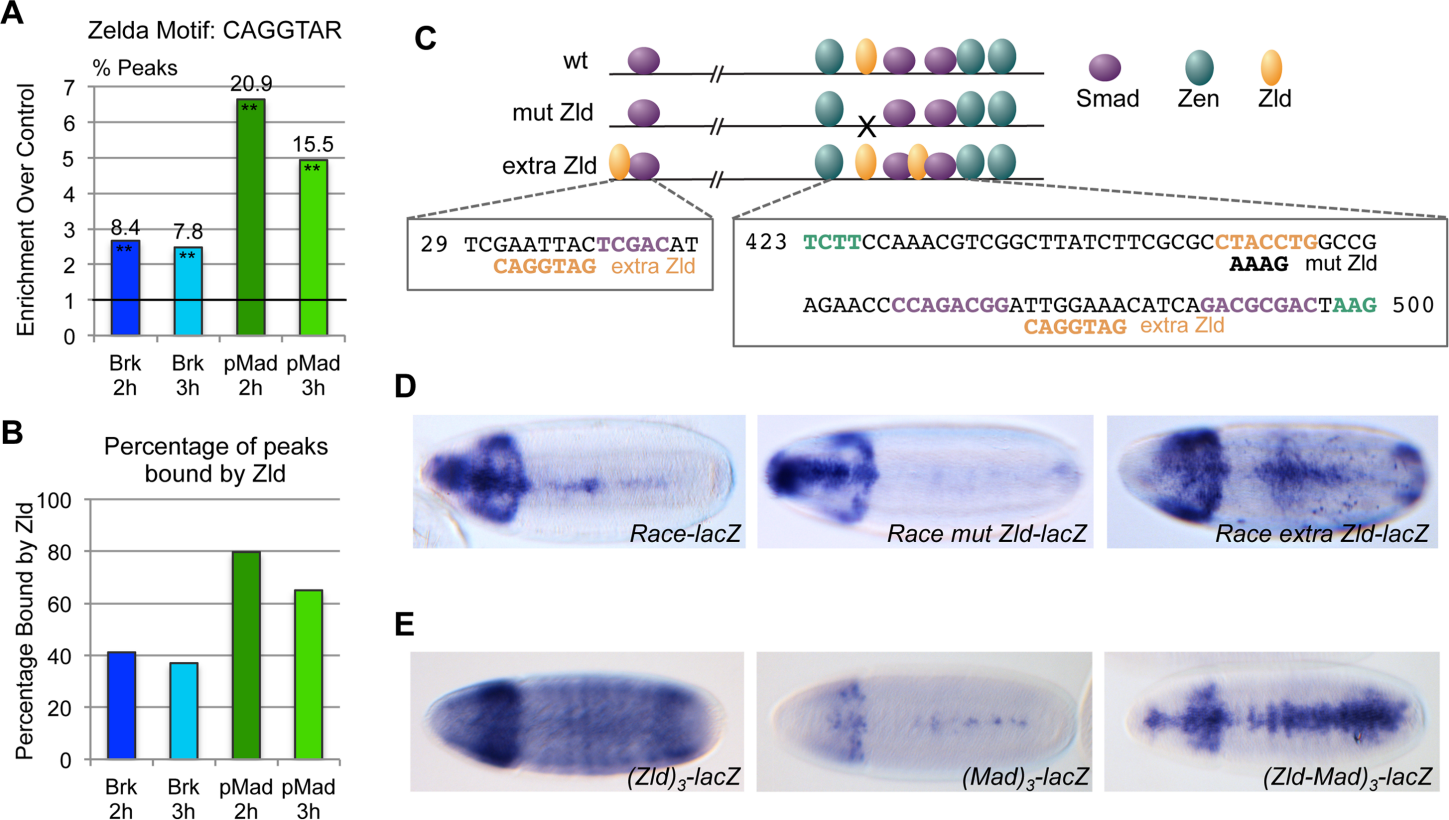
Zld is required for Dpp gradient interpretation. (A) Graph showing enrichment of two variants of the Zld binding motif in the indicated ChIP data sets relative to that of a set of housekeeping enhancers [20]. Other variants of the Zld binding site exist but are not included here. The line drawn at 1 represents no relative enrichment. The percentage of peaks harboring the motif in each data set is shown above the bar. Enrichment of the motif relative to the control set is significant at **P<0.01 based on Fisher’s exact two-tailed test. (B) Graph showing percentage of the peaks in each of the four data sets that overlap Zld regions identified by ChIP-seq [25]. (C) Cartoon showing the position of the Smad, Zen and Zld binding sites in the 533 bp wildtype and altered *Race* enhancer variants. Relevant binding site sequences are shown underneath with nucleotide shading as in the cartoon (purple—Smad, orange–Zelda, green–Zen). Only part of the sequences of the Zen binding sites is shown. The Smad binding sites are as described [26], although weak Smad binding has also been reported between the upstream Zen and Zelda binding sites [22]. (D) RNA in situ hybridization with a *lacZ* probe of embryos carrying a transgene with either a wildtype or altered *Race* enhancer, as shown in (C), upstream of a *lacZ* reporter gene. The transgenes are integrated at the same genomic site. (E) As in (D) except that *lacZ* expression is directed by 3 copies of either the Zld motif, Mad motif or the combined Zld-Mad motifs. Spacing between the three Zld or Mad motifs when tested in isolation is the same as when tested together.

We next performed *de novo* motif analysis on the four sets of ChIP peaks that we identified. This analysis identified the sites CAGGTAG and CAGGTAA across the data sets, which have previously been shown to be binding sites for Zld, a transcription factor with a central role in activation of the zygotic genome [23–25]. These Zld binding motifs are enriched in Brk and pMad bound regions, particularly the latter, relative to the control set of housekeeping enhancers [20] (Fig 6A), and there is extensive overlap between Zld binding regions identified by ChIP-seq [25] and the Brk and pMad binding regions (Fig 6B). To test the importance of Zld binding with respect to Dpp-dependent gene expression we mutated the Zld site in the *Race* enhancer (Fig 6C). Disruption of Zld binding leads to a loss of *Race* enhancer activity (Fig 6D). Adding two extra Zld binding sites to the enhancer around the Smad sites leads to an expanded expression pattern (Fig 6C and 6D), consistent with Zld being important for enhancer activity and output. To test this in another way, we used a multimerised binding site reporter assay. Three copies of the Zld binding site drive ubiquitous reporter gene expression although some striping is observed along the AP axis (Fig 6E). Multimerising three copies of a Mad binding site results in weak expression along the dorsal midline. In contrast, three copies of the Zld and Mad sites in tandem result in a distinct reporter gene expression pattern (Fig 6E), typical of an intermediate Dpp threshold response. Together these data suggest that Zld plays a key role in Dpp gradient interpretation.

### Extensive overlap with insulator proteins

**Fig 7:**
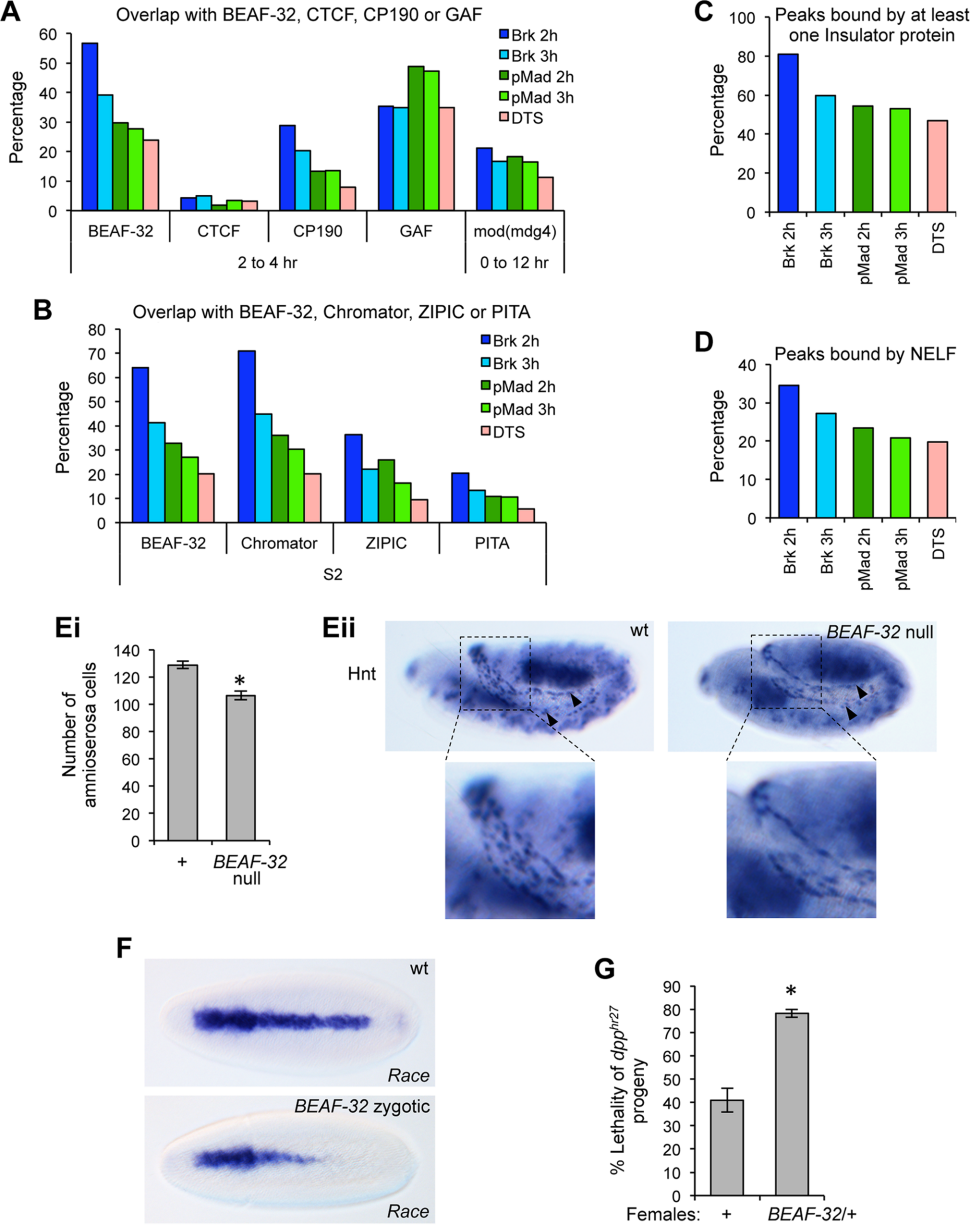
Insulator proteins bind to the pMad/Brk regions. (A, B) Graphs showing percentage overlaps between the pMad/Brk ChIP peaks and the indicated insulator proteins using ChIP data obtained from 2–4 h or 0–12 h embryos (A) or S2 cells (B), as labeled. In graphs (A-B), regions within the DTS data set that overlap with pMad/Brk peaks were removed prior to calculating the DTS-insulator binding protein overlap, in order to provide a cleaner comparison with the pMad/Brk data. However, this removal typically only lowers the overlap between the DTS data sets and the insulator binding proteins by less than 3%, with the exception of the Chromator-DTS overlap that is reduced by 9%. (C) Graph showing the percentage of ChIP peaks in each data set that bind at least one of the insulator proteins tested in (A, B) with the exception of GAF and using only the embryo data for BEAF-32. (D) Graph showing overlap with NELF-B and NELF-E binding based on ChIP data obtained for these factors in S2 cells. (E) Graph (Ei) showing the number of amnioserosa cells, as determined by Hnt staining, in wildtype and *BEAF*^*AB-KO*^ null embryos (n = 30 across 3 biological repeats, error bars are SEM, *p<0.01, two tailed t-test). Representative embryos are shown in (Eii), amnioserosa staining is indicated by black arrowheads, magnified views of the regions in the dashed squares are shown. (F) RNA in situ hybridizations showing a loss of posterior *Race* expression in embryos collected from *BEAF*^*AB-KO*^ heterozygous adults, compared to wildtype. (G) Graph showing the percentage lethality of *dpp*^*hr27*^ progeny from a cross of either wildtype or *BEAF32*^*ABKO*^/+ females to *dpp*^*hr27*^/CyO males (n = 3, >200 flies counted for each biological repeat, error bars show SEM, *p<0.05, two tailed t-test).

Other enriched motifs we identified across the data sets match the consensus sequences for BEAF-32, an insulator binding protein, and GAGA factor (GAF), a protein with diverse roles including insulator binding [27]. Analyses of the BEAF-32 or GAF ChIP-chip data from early *Drosophila* embryos [28, 29] reveal partial overlap with the pMad and Brk ChIP peaks (Fig 7A, S2 Dataset). To extend this finding, we also tested whether the CTCF, CP190 and Mod(mdg4) insulator proteins bind the pMad/Brk regions using available ChIP-chip data from embryos [27, 28]. These data (Fig 7A, S2 Dataset) show some overlap with CP190 and Mod(mdg4), but very little with CTCF. The percentage overlap between the pMad/Brk regions and those bound by insulator proteins is similar to or higher than that observed for enhancers bound by Dorsal, Twist and Snail (DTS) [12] (Fig 7A). In addition, the pMad/Brk ChIP regions show greater overlap with insulator proteins than that observed for Zld activated early enhancers [25] and a set of developmental enhancers active across embryogenesis [30] (S5A–S5D Fig).

Additional insulator proteins include Chromator, ZIPIC and PITA [29, 31]. As ChIP data from embryos is lacking for these factors, we made use of available data from *Drosophila* S2 cells [31, 32], with the BEAF-32 data from S2 cells [32] (Fig 7B) showing a similar overlap to that calculated based on the embryo BEAF-32 data (Fig 7A). Chromator, ZIPIC and PITA show partial overlap with pMad/Brk regions, at a higher level than that observed for the DTS enhancers (Fig 7B, S2 Dataset). We also determined the proportion of pMad/Brk regions that bind at least one insulator protein, initially including the insulator proteins shown in Fig 7A and 7B without GAF and only using the embryo data for BEAF-32. As shown in Fig 7C and S2 Dataset, 50–80% of peaks in the pMad/Brk data sets bind at least one insulator protein, similar to that observed for the DTS enhancers (Fig 7C). We excluded GAF from this analysis as GAF has roles additional to insulator binding, the percentage of pMad/Brk regions binding at least one insulator protein is higher when GAF is included (S5D Fig). In addition, NELF (negative elongation factor) that is associated with promoter proximal RNA polymerase II pausing has been reported to colocalize with BEAF-32 [33]. Using NELF-B/E ChIP-chip data [34], we found that NELF-B/E is bound to around one fifth of the pMad/Brk ChIP peaks, again comparable with NELF-B/E binding to the DTS enhancers (Fig 7D), but higher than for other enhancer sets (S5E Fig).

Recently, ‘Phantom Peaks’ have been described representing loci prone to non-specific enrichment in ChIP experiments [35]. Phantom Peaks are frequently found within 1kb of the promoters of active genes or overlapping HOT (highly occupied target) sites, and are present within many of the modENCODE data sets, including those relating to BEAF-32, GAF, CP190 and CTCF. As Phantom Peaks seem related to the use of antibodies in ChIP, DamID profiling data can be used to reassign some Phantom Peaks as real, based on their presence in the DamID data set [35]. Using this approach and BEAF-32 DamID data [36], we found that less than 10% of the pMad/BEAF-32 dual bound peaks are potential Phantom Peaks although the proportion is higher for Brk only at the 2–2.5 h time point (S5F Fig). Repeating this analysis for GAF revealed a similar trend, with a minority of GAF and pMad/Brk bound peaks potentially Phantom Peaks (S5F Fig), although GAF’s presence near promoters may relate to its function at promoters in displacing nucleosomes [37]. Together these data suggest that the majority of dual BEAF-32/pMad or GAF/pMad peaks are real.

To address the importance of BEAF-32 binding to the pMad/Brk enhancers we analyzed *BEAF*^*AB-KO*^ knockout embryos. These *BEAF*^*AB-KO*^ null flies are homozygous viable but sickly, especially in crowded conditions, and have poor fertility due to a BEAF-32 requirement during oogenesis [38], thus making it extremely difficult to collect enough null embryos for analysis. For this reason, we focused on counting the number of amnioserosa cells, the fate specified by peak Dpp signaling, in embryos from *BEAF*^*AB-KO*^ homozygous mutant adults. As shown in Fig 7Ei,ii, the BEAF-32 mutant embryos show a 20% reduction in the number of amnioserosa cells, compared to wildtype embryos. We also visualized *Race* expression in embryos from heterozygous *BEAF*^*AB-KO*^ adults, as these could be easily collected. There is a posterior loss of *Race* expression in approximately half of these embryos (Fig 7F), even though they receive a maternal dose of *BEAF-32*. In addition, genetic interaction analysis revealed that the lethality associated with the *dpp*^*hr27*^ allele in progeny from wildtype females [39] is significantly enhanced when females with only a single copy of BEAF-32 were tested (Fig 7G). Together these data suggest that insulator proteins facilitate Mad-dependent activation of some target genes.

## Discussion

We have combined RNA-seq, ChIP-seq and expression pattern data to identify the Dpp-responsive regulatory network in the early *Drosophila* embryo. Some of the Dpp target genes we have identified encode proteins with predicted functions compatible with the different events in the life of amnioserosa cells [40, 41], including cell division arrest–Toucan (Toc) [42] and Ana [43], changes in cell shape and adhesion–Bazooka (Baz) [44] and Rst [45], and programmed cell death during dorsal closure–Rst [46] and Scylla (Scyl) [47]. It will be interesting to precisely determine the timings at which these different early Dpp target genes are required. We have also identified new Dpp target genes for which a function is currently lacking. As the Dpp-responsive gene network appears relatively modest, given the tractability of the *Drosophila* embryo, this network therefore represents a relatively simple framework for future studies aimed at understanding how gene expression changes drive cell fate changes.

**Fig 8:**
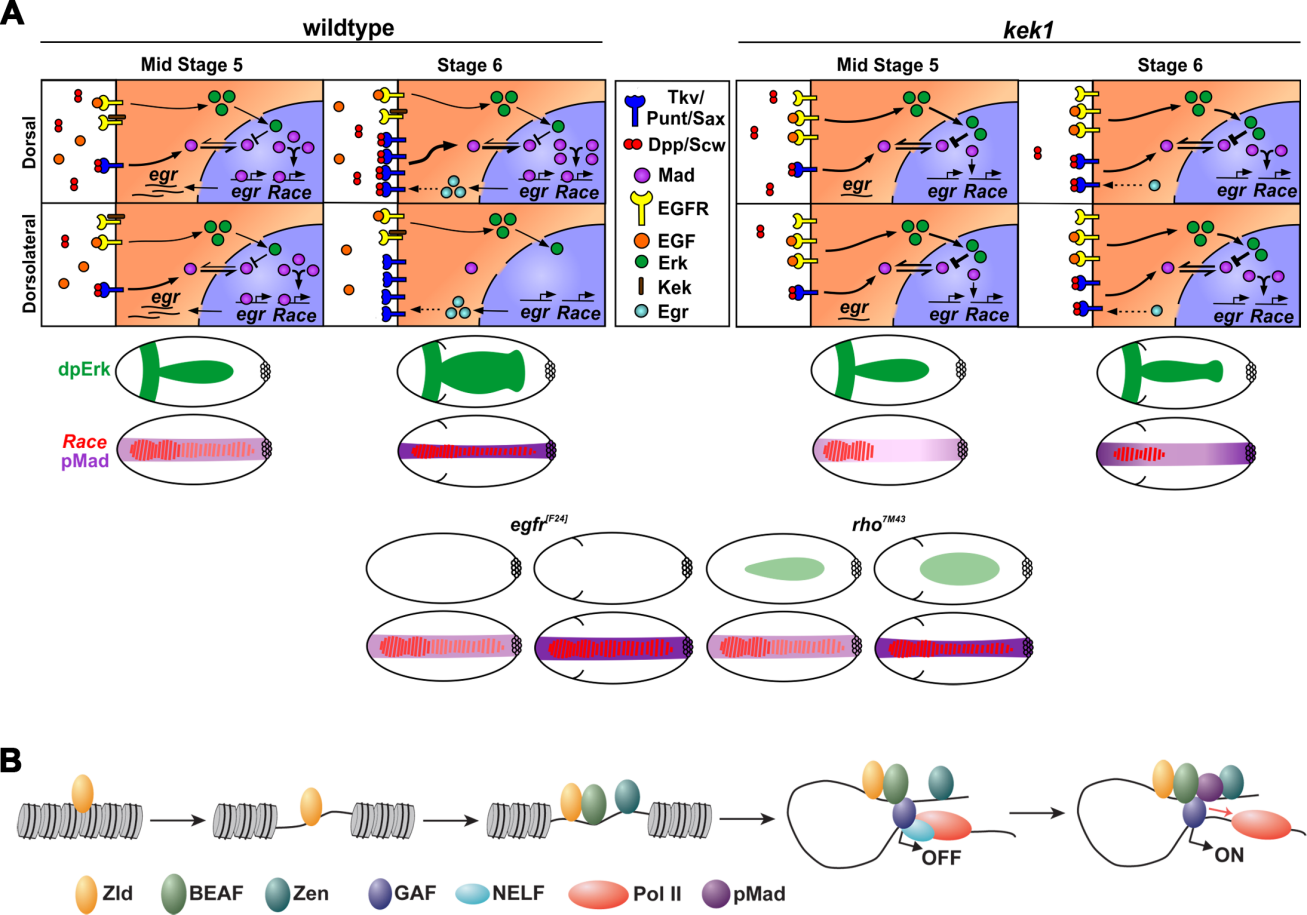
Models of pMad-dpERK antagonism and pMad activation. (A) Model showing the equilibrium between the positive and negative effects of Dpp and EGF, respectively, on pMad. The top panels show dpERK-pMad dynamics in a wildtype or *kek1* mutant cell at the dorsal midline (labeled Dorsal) compared to one positioned more dorsolaterally (Dorsolateral) at stages 5 and 6, as labelled, with the various proteins indicated in the key. The dpERK-pMad equilibrium is disrupted in *kek1* mutant embryos leading to reduced pMad. For each stage and genotype, the resulting dpERK, pMad and *Race* activation/expression domains within the whole embryo are depicted in the lower cartoons. Cartoons (stages as described above) are also shown for the EGF loss-of-function mutants tested. (B) Stepwise model of pMad-dependent activation, involving Zld, Zen and insulator binding proteins.

Many components of the EGF pathway are regulated by Dpp signaling, and our data support a model whereby EGF signaling regulates Dpp transduction through the controlled inhibition of Mad activity (Fig 8A). During embryonic stage 5, *rho* and *vein* are activated by Dpp signaling, enabling dorsal MAPK pathway activation [48]. Based on data from vertebrates showing that MAP kinase phosphorylation of Smad1 ultimately represses its activity by leading to its degradation [4], we propose that the activated dpERK promotes pMad degradation in the early embryo. Therefore, in our model the level of pMad is a balance between BMP receptor-mediated phosphorylation and linker phosphorylation initiated by dpERK (Fig 8A). In a wildtype embryo, this equilibrium establishes a nuclear pMad level that activates Dpp target genes including *egr*, with Egr then functioning via positive feedback to promote refinement of the pMad stripe at the dorsal midline, to allow activation of peak Dpp target genes (Fig 8A).

Embryos mutant for *kek1*, encoding an inhibitor of the EGF receptor [18], have a narrower domain of dpERK activation, which we attribute initially to the EGF ligand being sequestered by a greater number of uninhibited EGF receptors (Fig 8A). This narrower dpERK domain alters the pMad-dpERK equilibrium, so that pMad levels are dampened from stage 5. The lower pMad in *kek1* mutant embryos likely reduces expression of the positive target, *rho*, and leads to loss of *egfr* repression, which would contribute to the shrinking of the dpERK domain compared to wildtype embryos. As a result, *egr* activation and therefore positive feedback at stage 6 is compromised, leading to loss of refinement of the pMad stripe, and disrupted target gene expression (Fig 8A). Conversely, in *egfr* or *rho* mutant embryos, the loss/reduction in dpERK leads to expanded *Race* expression, consistent with the pMad-dpERK equilibrium being tipped in favour of higher pMad (Fig 8A). Together, these data suggest that the presence of both positive and negative regulators of EGF signaling renders the BMP network self-regulating, in that it can maintain a threshold of EGF signaling that is permissive for Dpp-dependent patterning. This Dpp-EGF interplay may also be important in other developmental contexts, such as the dorsal head region in the *Drosophila* embryo where overactivation of EGF signaling antagonises Dpp signaling [49].

Our ChIP data has allowed the identification of many Dpp dependent enhancers, with integration of our ChIP- and RNA-seq data suggesting that the majority of Dpp-responsive genes in the early embryo are direct targets. We have also shown that ~20% of the pMad/Brk genomic regions are bound by Dorsal, Twist and Snail, suggesting that the enhancers of some positive pMad targets are inactive in the mesoderm and neuroectoderm, not only due to Dorsal repression of the *dpp* gene, but also due to direct repression by Dorsal and/or Snail binding. These targets, including transcription factors and signaling pathway components, might be potent in transforming cell fate so that it is advantageous to have them under an additional level of negative control in the mesoderm and neuroectoderm. In addition, some dual Dpp/Dorsal targets include genes expressed in the neuroectoderm that may be activated by Dorsal in the neuroectoderm and repressed by Dpp signaling in the dorsal ectoderm, as described for *brk* [6].

Most of the pMad genomic regions we have identified are also bound by Zld and we show that Zld is required for *Race* enhancer activity, with extra Zld sites broadening the expression pattern. This latter effect may be through increased chromatin accessibility, as described for Dorsal activation [50]. Our data also show that Zen is important for peak and intermediate Dpp target gene expression, consistent with that reported for *Race*, *C15*, *egr* and *cv-2* previously [3, 21, 22]. *zen* is initially broadly expressed in the dorsal ectoderm but becomes restricted to the dorsal midline in response to Dpp signaling [15]. Given that *tup* and *ush* have broader expression patterns than the restricted *zen* pattern, it appears that the Zen prepattern is important for expression of at least some Dpp target genes.

We also found that insulator proteins bind to many of the pMad/Brk ChIP enhancers, including those for *Race*, *ana*, *egr*, *ush*, *Ama* and *pnr*. Consistent with this, a set of enhancers enriched for mesodermal enhancers has recently also been reported to show significant overlap with insulator proteins [51]. We also found insulator binding to other early embryonic enhancers (Zld, Dorsal), but to a lesser extent for a set of enhancers that are activated throughout embryogenesis, suggesting that insulator binding may be important for early activation. Analysis of *BEAF-32* null embryos showed a small but significant reduction in amnioserosa cell number and disrupted *Race* activation. This relatively mild phenotype, and the viability of the BEAF-32 null embryos, could reflect redundancy in insulator binding proteins. Recently, *Drosophila* Mad and Med have been found to colocalize with CTCF in tissue culture cells in the absence of Dpp signaling, but relocalize away from these regions when Dpp is present [52]. Consistent with this, we detect very poor overlap between regions bound by CTCF and the signaling activated form of Mad.

Insulators can form gene loops with active promoters in *Drosophila* [53, 54] and for some insulator binding proteins, including BEAF-32 and CP190, ChIP-seq has identified indirect binding peaks that have been attributed to long-range interactions. These include insulator-promoter interactions, with GAF-regulated paused RNA polymerase II (Pol II) at the promoter proposed to act as bait for the insulator interaction [55]. Such interactions may explain why we observe NELF binding to a subset of the pMad/Brk genomic regions. In addition, enhancer-promoter loops have been found to be developmentally stable and associated with Pol II pausing [51].

Based on our data and the findings described above, we propose a speculative model for Dpp-dependent activation whereby binding of the Zld pioneer transcription factor opens chromatin to allow recruitment of other proteins, including Zen (with the early broad expression important) and insulator proteins (Fig 8B). Insulator binding may promote looping of the enhancer to the promoter, with the majority of Dpp target genes harbouring paused Pol II [56, 57]. Subsequent recruitment of pMad, potentially aided by an interaction with Zen [22] and/or insulator proteins, would allow release of paused Pol II and gene activation. Parallels exist here with vertebrate Smads, as a potential role for pioneer transcription factors in Smad-dependent activation has been proposed [58]. In addition, Smad3 is recruited to the H19 Imprinting control region by CTCF, via a direct interaction shown between Smad3 and CTCF [59], suggesting that insulator proteins may also play a role in Smad-dependent activation in vertebrates.

## Materials and Methods

### Fly stocks

Fly stocks used were: *yw brk*^*M68*^/FM7c *ftz-lacZ* [6], *dpp*^*Hin37*^/GlaDp(2;2)DTD48, *dpp*^*hr27*^ *cn*^*1*^ *bw*^*1*^/CyO P{*dpp-P23*}, *pUASp*-*Tkv*^*QD*^ [10], *P{matα4-GAL-VP16}V2H*, *zen*^*7*^/TM3, *kek1*^*RA5*^/CyO, *kek1*^*RM2*^/CyO [18], *egfr*^*F24*^/CyO, *rho*^*7M43*^/TM3, *BEAF*^*AB-KO*^/CyO [38] and *y*^*67c23*^*w*^*118*^ which we used as wildtype. Embryo collections and in situ hybridization with digoxygenin-UTP-labelled probes were performed using standard protocols, or as previously described for fluorescent in situs and Hnt immunostaining [60]. Antibodies used were: anti-Digoxigenin-AP Fab fragments (1:250, Roche), sheep anti-digoxigenin (1:400, Roche), rabbit anti-dpMAPK (1:500, Sigma), rabbit anti-pSmad1/5/8 (gift from E. Laufer, 1:1000), mouse anti-Hnt (1:20, DSHB), donkey anti-rabbit-IgG-Alexa 488, donkey anti-sheep-IgG-Alexa 555 (both 1:500, Invitrogen) and goat anti-mouse-AP (Promega, 1:500). Immunostained embryos were mounted in Prolong Gold with DAPI (Invitrogen). Wildtype and mutant embryos were stained in parallel, confocal images were taken using the same settings and processed in the same manner. Genetic interactions were performed as described previously [39]. For the analysis of *Race*, *hnt* and *ush* expression, the width of the stripe of expression was counted as the number of expressing cells at ~0.5 embryo length.

### RNA-seq

Test and control embryos were collected from *P{matα4-GAL-VP16}V2H*/+; *P{UASp-Tkv*^*QD*^*}/+* and *P{matα4-GAL-VP16}V2H*/+ adults, respectively, at 3–3.5 h AEL. RNA was extracted from 30μl of *Drosophila* embryos per sample using Trizol (Invitrogen) and processed for RNA-seq using the Life Technologies RiboMinus Eukaryote kit and SOLiD Total RNA-Seq Kit. RNA-seq was performed on two biological replicates using the ABI SOLiD4 sequencing platform. Mapping of the RNA-seq reads to the *Drosophila* genome (BDGP R5/dm3) was performed using TopHat [61] and statistically significant differentially expressed transcripts were identified using Partek Genomic Suite (version 6.5) for transcriptome assembly and DESeq (v1.10.1) [62], with an adjusted p-value of <0.03 used as the statistical cut-off, as this cut-off included the majority of known Dpp target genes. GO term analysis was performed using the GeneCodis3 Web Software, with modular enrichment analysis [63]. RNA-seq data are available from Array Express with the accession number E-MTAB-1976.

### ChIP-seq

Embryo collections were performed as described previously [56]. The pMad antibody (pSmad1/5/8) was kindly provided by Ed Laufer and Thomas Jessell. Details of the Brk antibody generation and ChIP-seq method have been described [64]. Sequencing was performed on the ABI SOLiD4 sequencing platform. Sequence reads of 50bp in length were mapped to the *Drosophila* genome (BDGP R5/dm3) using BFAST (v0.6.4e) [65]; default parameters were used except from the ‘postprocess’ step that used the flag ‘-a 3’ that allows for unique best matches to be retained along with uniquely mapping reads. Peaks were identified using ChIPseeqer (v2) [66]; default parameters were used except the fragment length was set to 250bp, and the p-value cut off was set to 4 (the equivalent of 0.0001). The reads shown in the figures were normalized using a correction factor calculated as the mean of all median values divided by the per sample median value. For individual replicates the IP sample was normalised against the PI sample and viewed on the Integrated Genome Viewer v2.3.41 [67]. Identified binding regions are present in both ChIP-seq replicates and have a p-value of at least <0.0001. Where binding regions were identified with a cut-off of both p<0.0001 and p<0.00001, the higher resolution coordinates from the higher stringency data set were used. ChIP-seq data are available from Array Express with the accession numbers E-MTAB-1673 and E-MTAB-1674.

### RNA-seq and ChIP-seq integration

The Brk/pMad ChIP peaks were combined as a whole set (S2 Dataset, BMP list) or based on the factor across the two time points. When combining the 2–2.5 h and 3–3.5 h peak lists for a given factor (pMad or Brk), overlapping peaks were given the coordinates from the 3–3.5 h data. Similarly, when generating the combined Brk/pMad ChIP peak list, pMad 3–3.5 h coordinates were retained where appropriate. Significant association between these Brk/pMad ChIP peak sets and the differentially expressed genes (identified by RNA-seq and mining Flyexpress) was tested using RnaChipIntegrator (Briggs PJ, Donaldson IJ, Zeef LAH. RnaChipIntegrator, available at: https://github.com/fls-bioinformatics-core/RnaChipIntegrator version 0.3.2). For the ‘gene to peak’ analysis, genes located within a given distance of ChIP peak summits (5kb, 10kb, 20kb or 40kb) were counted for (i) differentially or (ii) non-differentially expressed genes. To test the statistical significance of whether differentially expressed genes were more likely to be associated with ChIP peaks than non-differentially expressed genes, these counts were used in binomial and Pearson's chi-square tests. For the ‘peak to gene’ analysis, ChIP peak summits located within a given distance of the transcription start site of differentially expressed genes were determined using the RnaChipIntegrator software. The gene set referred to as ‘confirmed upregulated’ includes the genes confirmed as being expressed in the early embryo (labeled as Y in S1 Dataset) and the set identified based on expression from Flyexpress (S1 Dataset).

### Integration of Brk/pMad ChIP-seq data with other data sets

Overlapping genomic intervals were identified using the intersect tool, as part of the BEDTools suite (v 2.25.0), whilst including the -u parameter and reporting a minimum of 1 bp overlap [68]. Percentage overlap was calculated as the number of pMad/Brk intervals overlapping insulator binding protein or NELF intervals [27–29, 31, 32, 34] divided by the total number of pMad/Brk ChIP peaks. A similar analysis was performed with embryonic Dorsal, Twist and Snail (DTS) ChIP-chip [12] or Zelda ChIP-seq data (combined stages) [25], in addition to a set of embryonic developmental enhancers [30]. In the case of the DTS data, these genomic regions include the high confidence genomic regions bound by a combination of: 1) Dorsal, Twist and Snail or 2) Twist and Snail [12]. Prior to comparison with these DTS, Zelda or embryonic enhancers, overlapping pMad/Brk ChIP regions were removed from these data. Where several sets of data were available for a single factor, interval files were concatenated and merged using the BEDTools merge tool [68].

### Overlap with Phantom Peaks

Candidate Phantom Peaks were identified by intersecting Phantom Peaks data [35] with the BEAF-32 or GAF regions that overlap pMad/Brk peaks. These potential Phantom Peaks were then subdivided into those: 1) present in the BEAF32 or GAF DamID data [36], reassigned as “Real” peaks, or 2) those absent, ie remaining as potential Phantom Peaks. DamID overlaps with the test Phantom Peaks were identified as described above, after converting the intervals to dm3, using the UCSC lift-over tool [69]. Overlaps with the DTS data were analyzed in the same way.

### Motif discovery

Weeder v1.4.2 motif discovery analysis [70] was performed on 250bp regions centered at the binding region summits. The percent of peaks containing the motif in 125bp either side of the summit was calculated. The control 250bp regions were from housekeeping enhancers [20].

### Enhancer cloning

pMad/Brk binding regions were cloned into plasmid pLacZattB using the primer sequences shown in S1 Table. This table also shows the motif primers and primers used to alter Zld sites within the *Race* enhancer. All transgenes were inserted into cytosite 86Fb.

## Supporting Information

S1 Fig GO-term functional ontology. GO-term categories, with associated p-values, for positive and negative Dpp targets genes identified by RNA-seq and FlyExpress data-mining.
(TIF)

S2 Fig Integration of the RNA- and ChIP-seq data. (A) Graph (Ai) showing the percentage of Brk, pMad or BMP (all Brk and pMad peaks combined) ChIP peaks that have a differentially expressed gene within the indicated distances ranging from 5 to 40kb. Graph (Aii) shows the probability that the differentially expressed genes are more likely to be closer to the ChIP peaks than non-differentially expressed genes. Higher significance is observed for the pMad than the Brk peaks, consistent with Brk only regulating a subset of Dpp target genes in the embryo. (B) Graph showing the percentage of either (Bi) total differentially expressed genes or (Bii) those confirmed to be positive targets in the early embryo (see S1 Dataset: RNA-seq genes annotated as Y plus the Flyexpress targets) present within the indicated distances from the ChIP peaks. Matched peaks and genes from (A) and (B) are listed in S3–S5 Datasets. (C) Venn diagrams showing the overlap between Dorsal, Twist and Snail (DTS) genomic regions and those bound by Brk or pMad at 3–3.5 h, or the complete BMP peak list that spans both time points.
(TIF)

S3 Fig Dpp-EGF cross-talk. (A) Expression of the *egfr* and *aos* genes detected by RNA in situ hybridizations of cellularized embryos (lateral views) that are either wildtype or *brk* mutant, as labeled. Arrowheads indicate expanded expression of the genes in *brk* mutant embryos. (B) Expression of the *egfr*, *aos*, *rho* and *vein* genes in wildtype or *dpp* mutant embryos, as detected by RNA in situ hybridization. Embryos are dorsal views at the onset of gastrulation. Expression of *egfr* and *aos* is detected at the dorsal midline in *dpp* mutant embryos (arrowheads), but not in wildtype embryos. In contrast, expression of *rho* and *vein* observed in the dorsal ectoderm in wildtype embryos is lost in *dpp* mutant embryos (arrowheads), as expected for these positive Dpp targets. For *rho* and *vein*, the expression of these genes in the presumptive neuroectoderm is also detected, both in wildtype and *dpp* mutant embryos.
(TIF)

S4 Fig Mad, Med and Brk motif enrichment. (A-C) Graphs showing enrichment of Mad [7], Medea [5] and Brk [19] motifs in the four sets of ChIP-seq binding regions compared to a control set of housekeeping enhancers [20]. In all graphs the line is drawn at 1 represents no relative enrichment. The percentage of peaks in each data set that harbor the motif is shown above each bar. Enrichment of the motif relative to the control set is significant at *P<0.05 and **P<0.01 based on Fisher’s exact two-tailed test. (A-B) The Mad and Med motifs occur more frequently within the pMad ChIP peaks compared to the Brk peaks. (C) The Brk motif is found within at least one third of the pMad and Brk binding regions, although the fold enrichment relative to the control set is higher in the pMad regions consistent with Mad also being able to bind to the Brk site [5]. Of the enhancers analyzed in Fig 2, Brk motifs are associated with intermediate and broadly expressed genes, but not peak targets. This is in agreement with Brk repression potentially being important for establishing the expression limits of intermediate and broad Dpp target genes, but not relevant to regulation of peak Dpp targets far from the Brk source [71].
(TIF)

S5 Fig Insulator binding to pMad/Brk regions. (A, B) Graphs show the percentage of BMP (combined pMad and Brk ChIP regions), DTS [12], Zelda [25] and Embryonic [30] enhancers that bind the indicated insulator proteins, based on ChIP data sets for these proteins from either embryos (A) or S2 cells (B). (C) Graph shows the percentage of peaks within the indicated enhancer sets that bind at least one insulator protein, excluding GAF and based on the embryonic (not S2 cell) data for BEAF-32. (D) As in (C) except GAF data are included for the combined BMP regions (left) and separate pMad and Brk data sets (right). (E) Overlap between NELF proteins and the different enhancer sets, based on NELF-B and NELF-E ChIP-chip data from S2 cells [34]. In graphs (A-E), pMad/Brk enhancers present in the DTS, Zelda and Embryonic data sets were removed from these data sets before calculating their insulator protein overlap, to allow a cleaner comparison to the BMP enhancers. With the exception of the Chromator-DTS overlap that is reduced by 9%, this removal lowers the percentage overlap between the DTS, Zelda and Embryonic data sets and the insulator binding proteins by less than 3%. (F) Graph shows the percentage of pMad/Brk and BEAF-32/GAF overlapping regions at the two time points, labeled as peaks (peach bars). A subset of these peaks was classified as potential Phantom Peaks, based on their overlap with a Phantom Peaks list [35] from which regions present in the relevant BEAF-32/GAF DamID data set [36] had been removed. Subtracting these potential Phantom Peaks from the total pMad/Brk and BEAF-32/GAF dual bound regions (peaks) gives the percentage labeled as “real” peaks.
(TIF)

S1 Dataset Genes differentially expressed in response to ectopic Dpp signaling. Table shows a list of genes identified by RNA-seq, separated as upregulated and downregulated, with each set sorted on adjusted p-value. The *tkv* and *w* genes present in the *pUASp*-*Tkv*^*QD*^ transgene are shaded grey, known Dpp target genes are highlighted in red. The genes identified from Flyexpress based on dorsal ectoderm expression are also listed in a separate sheet.
(XLSX)

S2 Dataset ChIP-seq data for pMad and Brk at 2–2.5 h and 3–3.5 h. Table shows a list of the ChIP-seq peaks for pMad or Brk at each time point. Overlaps of these peak regions with those identified for pMad or Brk at the other time points, Dorsal, Twist and Snail (DTS), Zld, NELF-B/E and the different insulator binding proteins (data are from embryos unless labeled otherwise) are indicated. The BMP sheet shows the combined pMad/Brk ChIP-seq data set.
(XLSX)

S3 Dataset Integration of the RNA-seq data with the combined BMP (pMad/Brk) ChIP-seq data. Data show integration over distances of 5, 10, 20 and 40 kb. Start and end coordinates relate to the peak summits. The ‘gene to peak’ data show genes within the indicated distance listed against the ChIP peaks, whereas the reciprocal matching is shown for the ‘peak to gene’ data.
(XLSX)

S4 Dataset Integration of the RNA-seq data with the pMad ChIP-seq data. As described for S3 Dataset except the data relate to the pMad ChIP-seq data.
(XLSX)

S5 Dataset Integration of the RNA-seq data with the Brk ChIP-seq data. As described for S3 Dataset except the data relate to the Brk ChIP-seq data.
(XLSX)

S1 Table Sequences of the primers used in this study. (DOCX)
