# Supplementary figures and images for: Regulation of the BMP Signaling-Responsive Transcriptional Network in the Drosophila Embryo
Source: PLoS Genet. 2016 Jul 5;12(7):e1006164. doi: 10.1371/journal.pgen.1006164 (PMC4933369; doi:10.1371/journal.pgen.1006164)

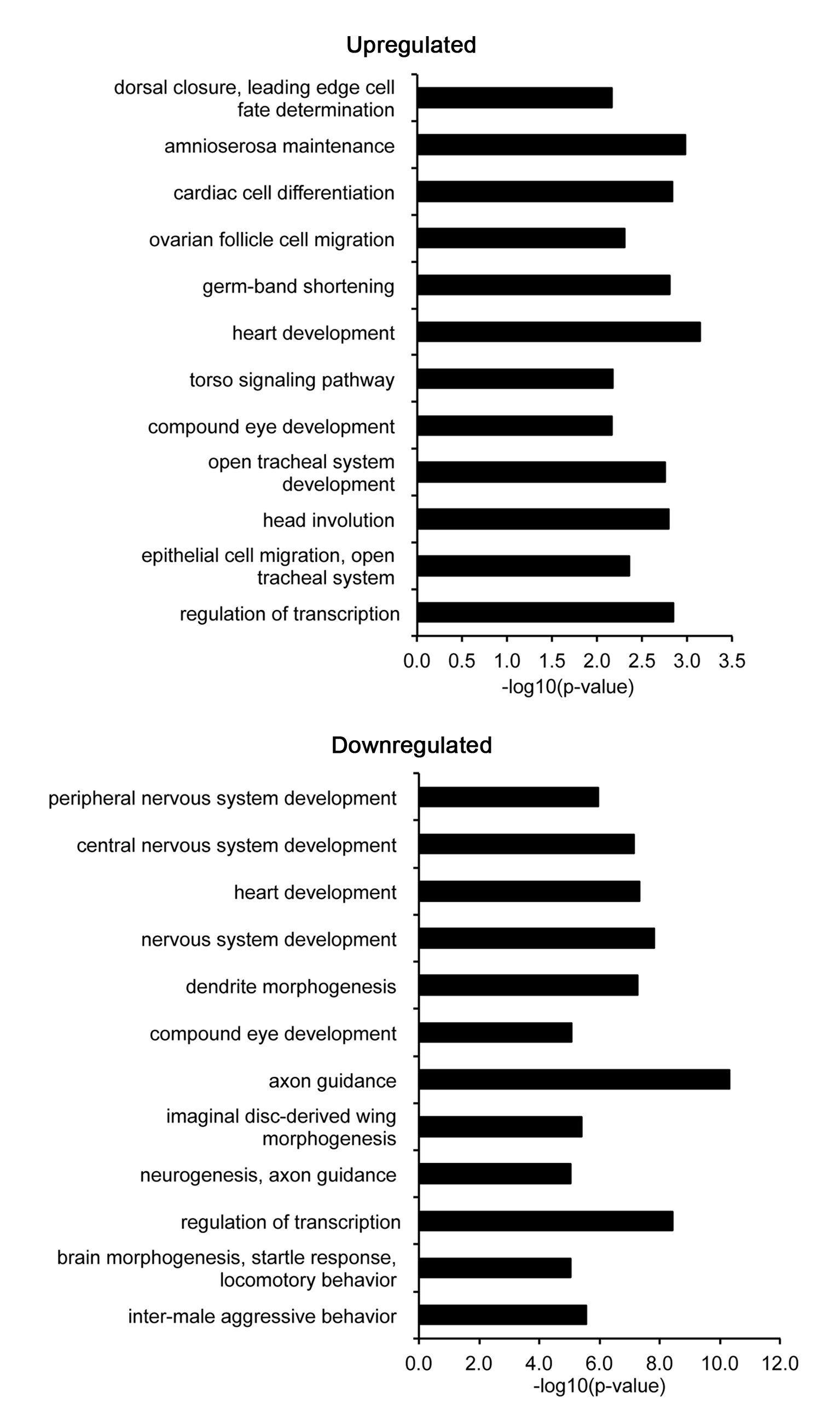

Supplement: S1 Fig — GO-term categories, with associated p-values, for positive and negative Dpp targets genes identified by RNA-seq and FlyExpress data-mining. (TIF) [file pgen.1006164.s001.tif]

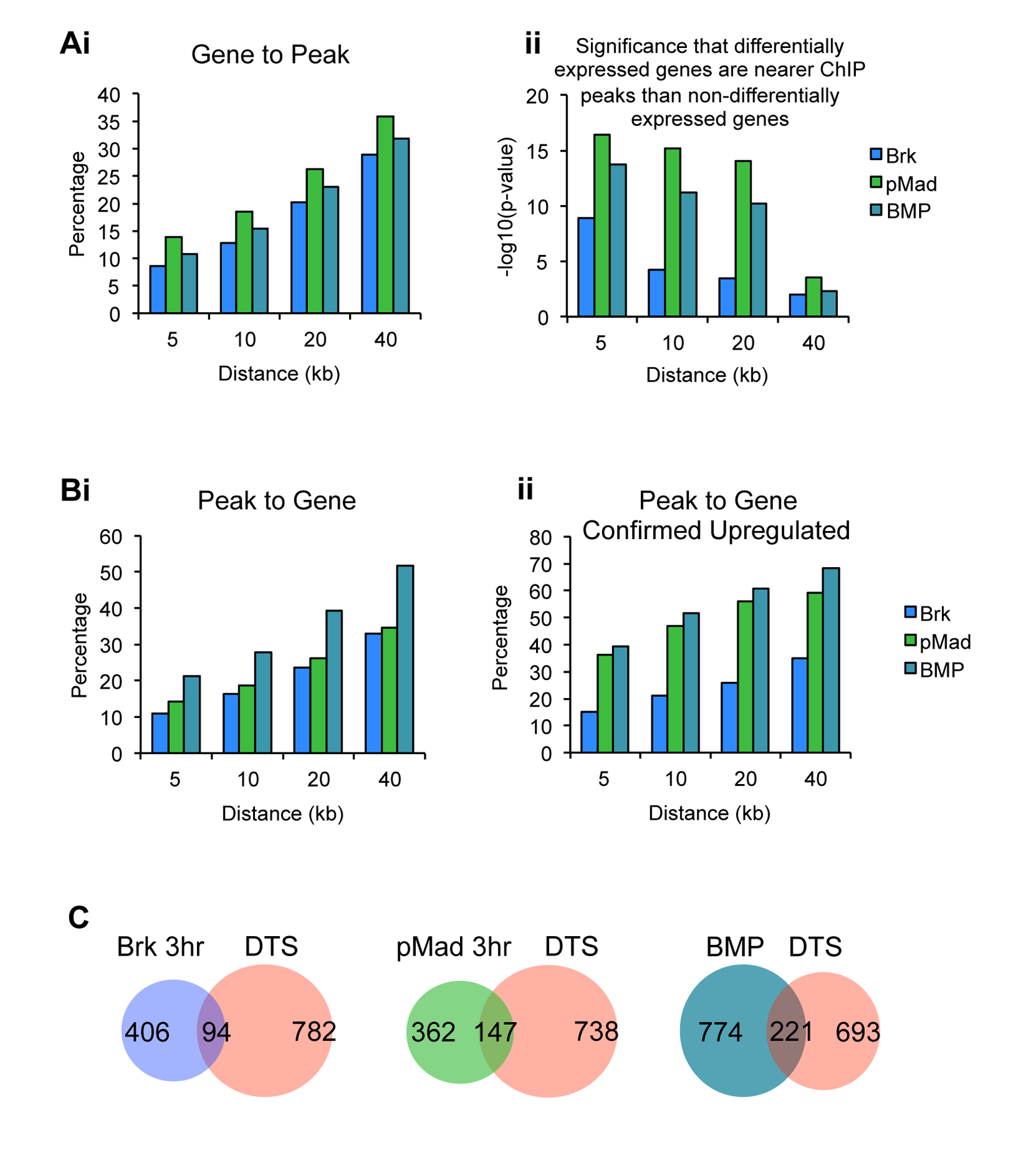

Supplement: S2 Fig — (A) Graph (Ai) showing the percentage of Brk, pMad or BMP (all Brk and pMad peaks combined) ChIP peaks that have a differentially expressed gene within the indicated distances ranging from 5 to 40kb. Graph (Aii) shows the probability that the differentially expressed genes are more likely to be closer to the ChIP peaks than non-differentially expressed genes. Higher significance is observed for the pMad than the Brk peaks, consistent with Brk only regulating a subset of Dpp target genes in the embryo. (B) Graph showing the percentage of either (Bi) total differentially expressed genes or (Bii) those confirmed to be positive targets in the early embryo (see S1 Dataset: RNA-seq genes annotated as Y plus the Flyexpress targets) present within the indicated distances from the ChIP peaks. Matched peaks and genes from (A) and (B) are listed in S3–S5 Datasets. (C) Venn diagrams showing the overlap between Dorsal, Twist and Snail (DTS) genomic regions and those bound by Brk or pMad at 3–3.5 h, or the complete BMP peak list that spans both time points. (TIF) [file pgen.1006164.s002.tif]

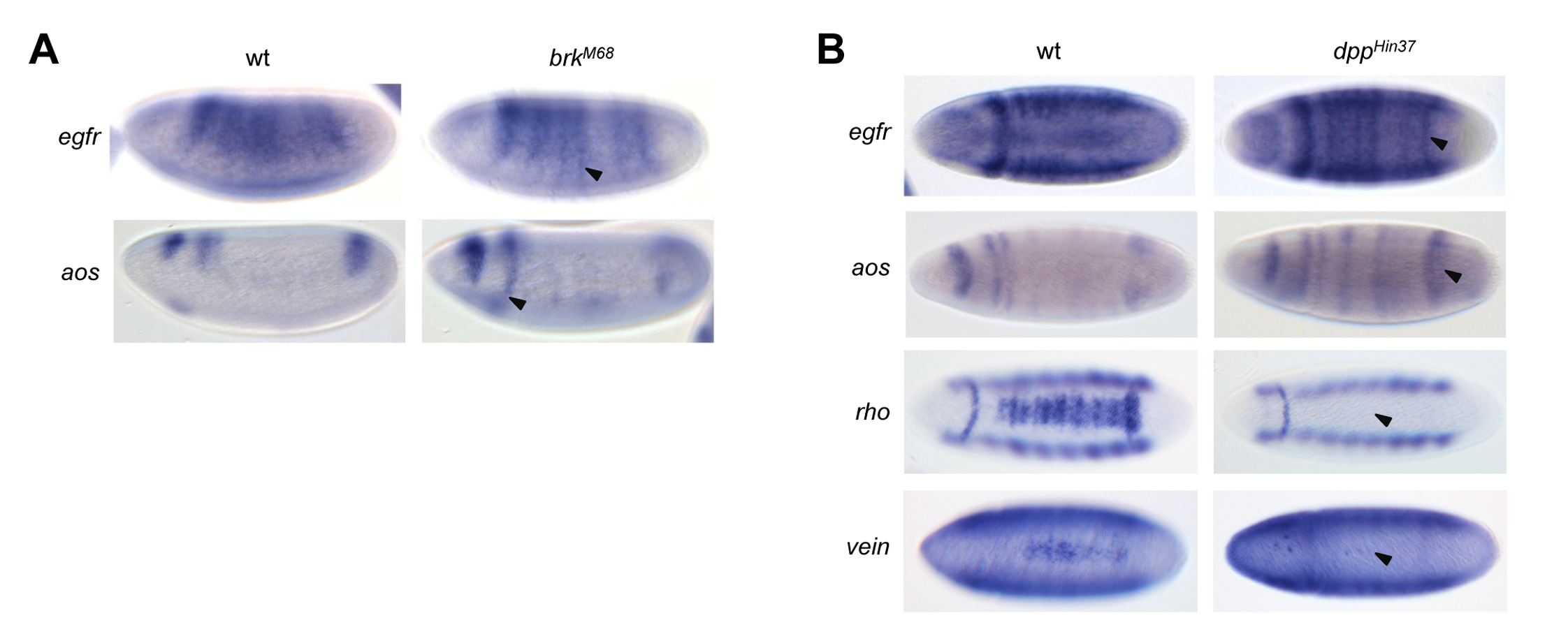

Supplement: S3 Fig — (A) Expression of the egfr and aos genes detected by RNA in situ hybridizations of cellularized embryos (lateral views) that are either wildtype or brk mutant, as labeled. Arrowheads indicate expanded expression of the genes in brk mutant embryos. (B) Expression of the egfr, aos, rho and vein genes in wildtype or dpp mutant embryos, as detected by RNA in situ hybridization. Embryos are dorsal views at the onset of gastrulation. Expression of egfr and aos is detected at the dorsal midline in dpp mutant embryos (arrowheads), but not in wildtype embryos. In contrast, expression of rho and vein observed in the dorsal ectoderm in wildtype embryos is lost in dpp mutant embryos (arrowheads), as expected for these positive Dpp targets. For rho and vein, the expression of these genes in the presumptive neuroectoderm is also detected, both in wildtype and dpp mutant embryos. (TIF) [file pgen.1006164.s003.tif]

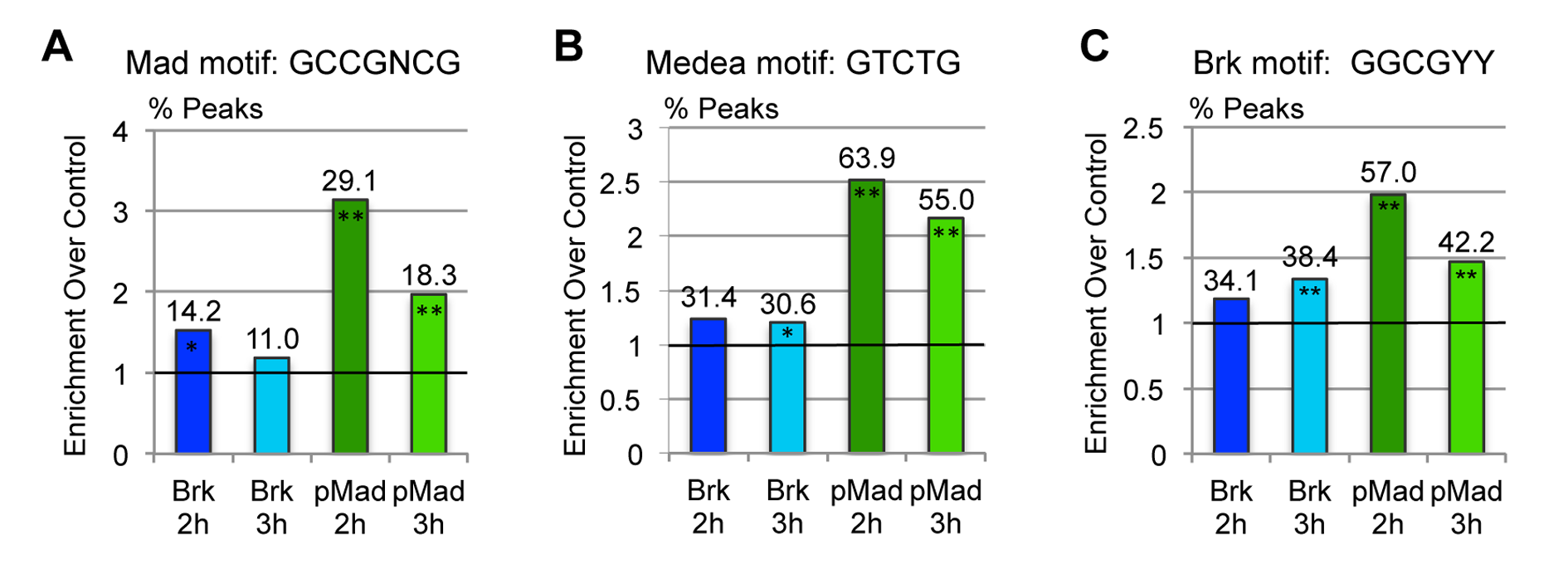

Supplement: S4 Fig — (A-C) Graphs showing enrichment of Mad [7], Medea [5] and Brk [19] motifs in the four sets of ChIP-seq binding regions compared to a control set of housekeeping enhancers [20]. In all graphs the line is drawn at 1 represents no relative enrichment. The percentage of peaks in each data set that harbor the motif is shown above each bar. Enrichment of the motif relative to the control set is significant at *P<0.05 and **P<0.01 based on Fisher’s exact two-tailed test. (A-B) The Mad and Med motifs occur more frequently within the pMad ChIP peaks compared to the Brk peaks. (C) The Brk motif is found within at least one third of the pMad and Brk binding regions, although the fold enrichment relative to the control set is higher in the pMad regions consistent with Mad also being able to bind to the Brk site [5]. Of the enhancers analyzed in Fig 2, Brk motifs are associated with intermediate and broadly expressed genes, but not peak targets. This is in agreement with Brk repression potentially being important for establishing the expression limits of intermediate and broad Dpp target genes, but not relevant to regulation of peak Dpp targets far from the Brk source [71]. (TIF) [file pgen.1006164.s004.tif]

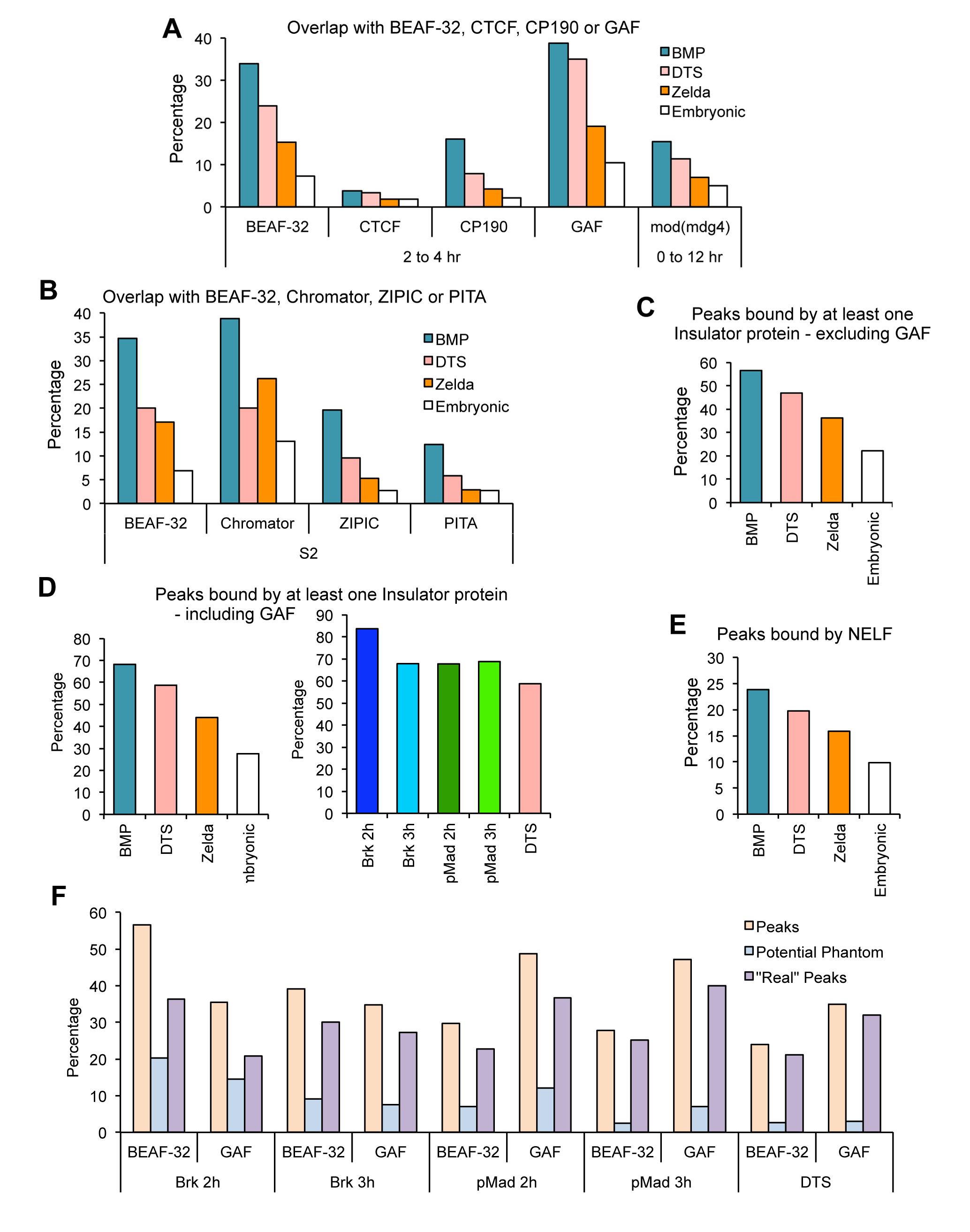

Supplement: S5 Fig — (A, B) Graphs show the percentage of BMP (combined pMad and Brk ChIP regions), DTS [12], Zelda [25] and Embryonic [30] enhancers that bind the indicated insulator proteins, based on ChIP data sets for these proteins from either embryos (A) or S2 cells (B). (C) Graph shows the percentage of peaks within the indicated enhancer sets that bind at least one insulator protein, excluding GAF and based on the embryonic (not S2 cell) data for BEAF-32. (D) As in (C) except GAF data are included for the combined BMP regions (left) and separate pMad and Brk data sets (right). (E) Overlap between NELF proteins and the different enhancer sets, based on NELF-B and NELF-E ChIP-chip data from S2 cells [34]. In graphs (A-E), pMad/Brk enhancers present in the DTS, Zelda and Embryonic data sets were removed from these data sets before calculating their insulator protein overlap, to allow a cleaner comparison to the BMP enhancers. With the exception of the Chromator-DTS overlap that is reduced by 9%, this removal lowers the percentage overlap between the DTS, Zelda and Embryonic data sets and the insulator binding proteins by less than 3%. (F) Graph shows the percentage of pMad/Brk and BEAF-32/GAF overlapping regions at the two time points, labeled as peaks (peach bars). A subset of these peaks was classified as potential Phantom Peaks, based on their overlap with a Phantom Peaks list [35] from which regions present in the relevant BEAF-32/GAF DamID data set [36] had been removed. Subtracting these potential Phantom Peaks from the total pMad/Brk and BEAF-32/GAF dual bound regions (peaks) gives the percentage labeled as “real” peaks. (TIF) [file pgen.1006164.s005.tif]
