# Supplementary material for: Regulation of the BMP Signaling-Responsive Transcriptional Network in the Drosophila Embryo
Source: PLoS Genet. 2016 Jul 5;12(7):e1006164. doi: 10.1371/journal.pgen.1006164 (PMC4933369; doi:10.1371/journal.pgen.1006164)
Supplement: S1 Table — (DOCX) [file pgen.1006164.s011.docx]

| **Enhancer** | **Primer sequences** |
| --- | --- |
| tup1 | CCCTCTGCTCCGCCGATCTTCGCC |
|  | TTATACGCTTTTAATTAGCGCTAA |
| tup2 | TAGAAATTTTCCCAGATATCTGTT |
|  | AGAGGGGGTAAGAGATGATGCACT |
| Doc1-1 | TTAGGGTTGTCCATGATTTATTGA |
|  | AAGCAGATCTCTGTTCAGATACAC |
| Doc1-2 | GATGATCATCACAAAGAGCGGCCG |
|  | GCCGAGAAAGTGACTGTTATCGCG |
| lov | GAAGGATTCGCTTTCGTTGCTATA |
|  | AAGTGCTAAAATCTGGCGAATAGC |
| rst1 | GGGCGATCGTAAAAATGTCAAGCG |
|  | AGACAACGACCCCCACAAATGGCC |
| rst2 | CGTCTCCGTTCACAAATTATAGAT |
|  | CTGCCCGAATACCCCGGATACCC |
| ana1 | ACAACTGCAGAATGCATCGAATTCC |
|  | TGAGTCACGCATGCGCAGAGGCAG |
| ana2 | GCGTGACTCACGCATGAACTCAAA |
|  | CGTTCTTGTTTATGTTTATGTTTA |
| zen2 | TTAGATACAAACGCAGACGCAGAC |
|  | TATTTAACTATATAAAAATTGTCT |
| net | CATCGCACTGTATAGCATGCCATG |
|  | GTGCATGCGCTTTGAGTTTGTTAG |
| Ama | CACAGATACAAAATACAGATAGAG |
|  | AGTAAATAAGAAGGAAAGAATTCT |
| cv2 | GTTCGGCGTTTGCTTGGCGCCACT |
|  | AAGCGCAGCGGGCGAAAATAGATTT |
| shn | GTGCACGGCAAGAGCCACGAAGA |
|  | GTACAGTGAAACTCCGCCATGGG |
| CG15480 | GACCATTGTGACTAAGCAG |
|  | CTCCTGGCGACGCTTTCGT |
| pnr | TGAATAGAAAAGTAAGAAAATGTA |
|  | TTCCGGTGCTTTTGTCTCTGTTTT |
| RACE mut Zld | CTGGGGTTCTCGGCCA**CTTT**GGCGCGAAGATAAGCC |
|  | GGCTTATCTTCGCGCC**AAAG**TGGCCGAGAACCCCAG |
| RACE extra Zld1 | TACATTCGAAATGTAAAATGTCGA**CTACCTG**GAATAACCCGCCGATGAGACAACAATC |
|  | GATTGTTGTCTCATCGGCGGGTTATTC**CAGGTAG**TCGACATTTTACATTTCGAATGTA |
| RACE extra Zld2 | GCTTAGTCGCGTCTGATG**CTACCTG**TCCGTCTGGGGTTCTCGG |
|  | CCGAGAACCCCAGACGGA**CAGGTAG**CATCAGACGCGACTAAGC |
| (Mad w/o Zld)3 | GGCGCCACGATACGATTTTA x 3 |
|  | TAAAATCGTATCGTGGCGCC x 3 |
| (Zld w/o Mad)3 | ACGATGACGATCAGGTAGTA x 3 |
|  | TACTACCTGATCGTCATCGT x 3 |
| (Mad-Zld)3 | GGCGCCACGATCAGGTAGTA x 3 |
|  | TACTACCTGATCGTGGCGCC x 3 |
